# Supplementary material for: Substantial Impairment of Quality of Life during COVID-19 Pandemic in Patients with Advanced Rectal Cancer
Source: Healthcare (Basel). 2022 Aug 11;10(8):1513. doi: 10.3390/healthcare10081513 (PMC9407989; doi:10.3390/healthcare10081513)
Supplement: Supplementary file 1 [file healthcare-10-01513-s001.zip › healthcare-1826963-supplementary.pdf]

Supplementary Materials:

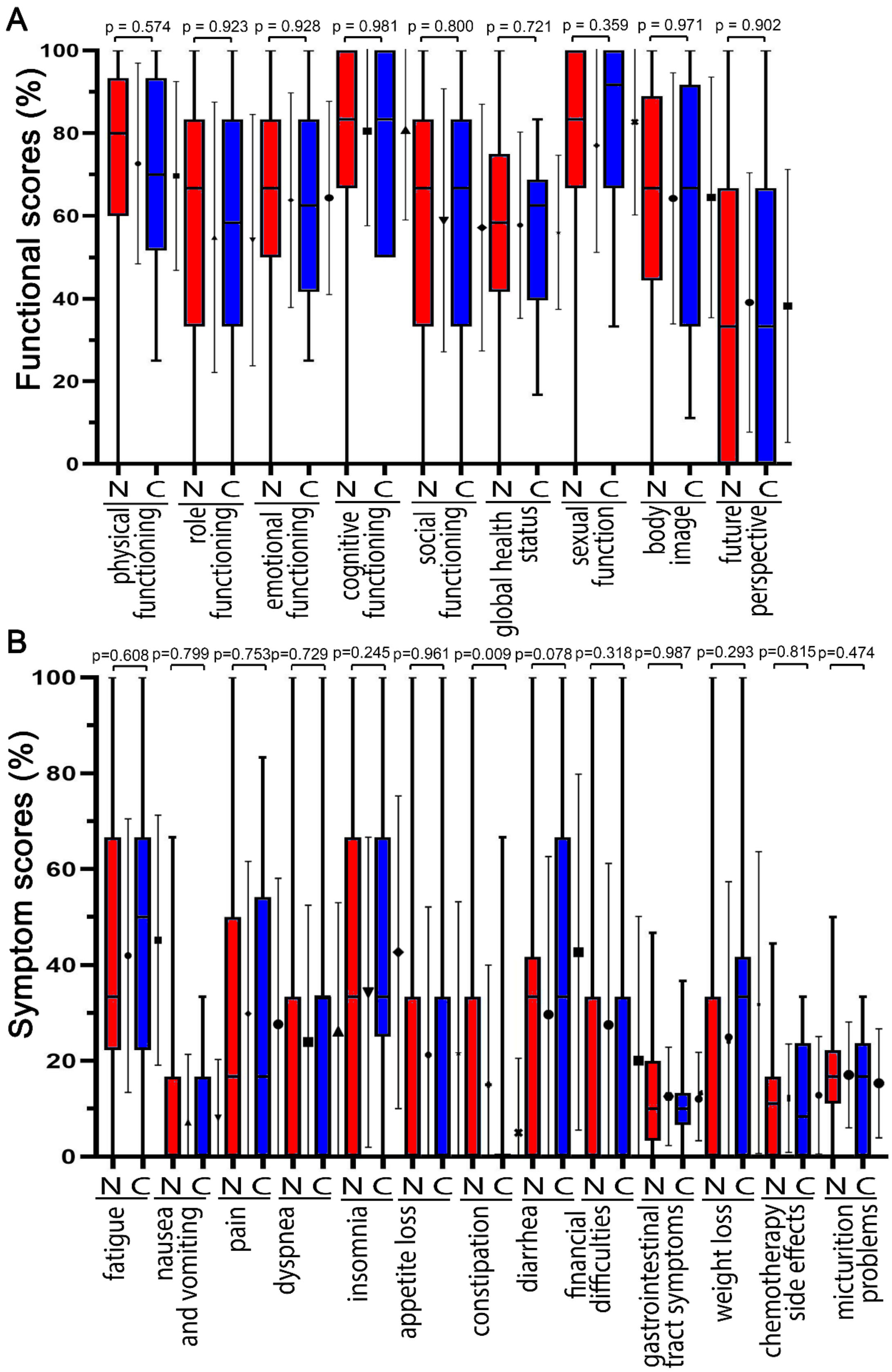

Figure S1. *The functional and symptom score distribution of prior to COVID-19 pandemic (N = non COVID) compared with during COVID-19 pandemic (C = during COVID pandemic). Scores were surveyed on day 70 (5 weeks) after radiochemotherapy.* ***(A)*** *Functional scores are: physical functioning, role functioning, emotional functioning, cognitive functioning, social functioning, global health status, sexual function, body image, and future perspective.* ***(B)*** *Symptom scores are: fatigue, nausea and vomiting, pain, dyspnea, insomnia, appetite loss, constipation, diarrhea, financial difficulties, gastrointestinal tract symptoms, weight loss, chemotherapy side effects, and micturition problems.*

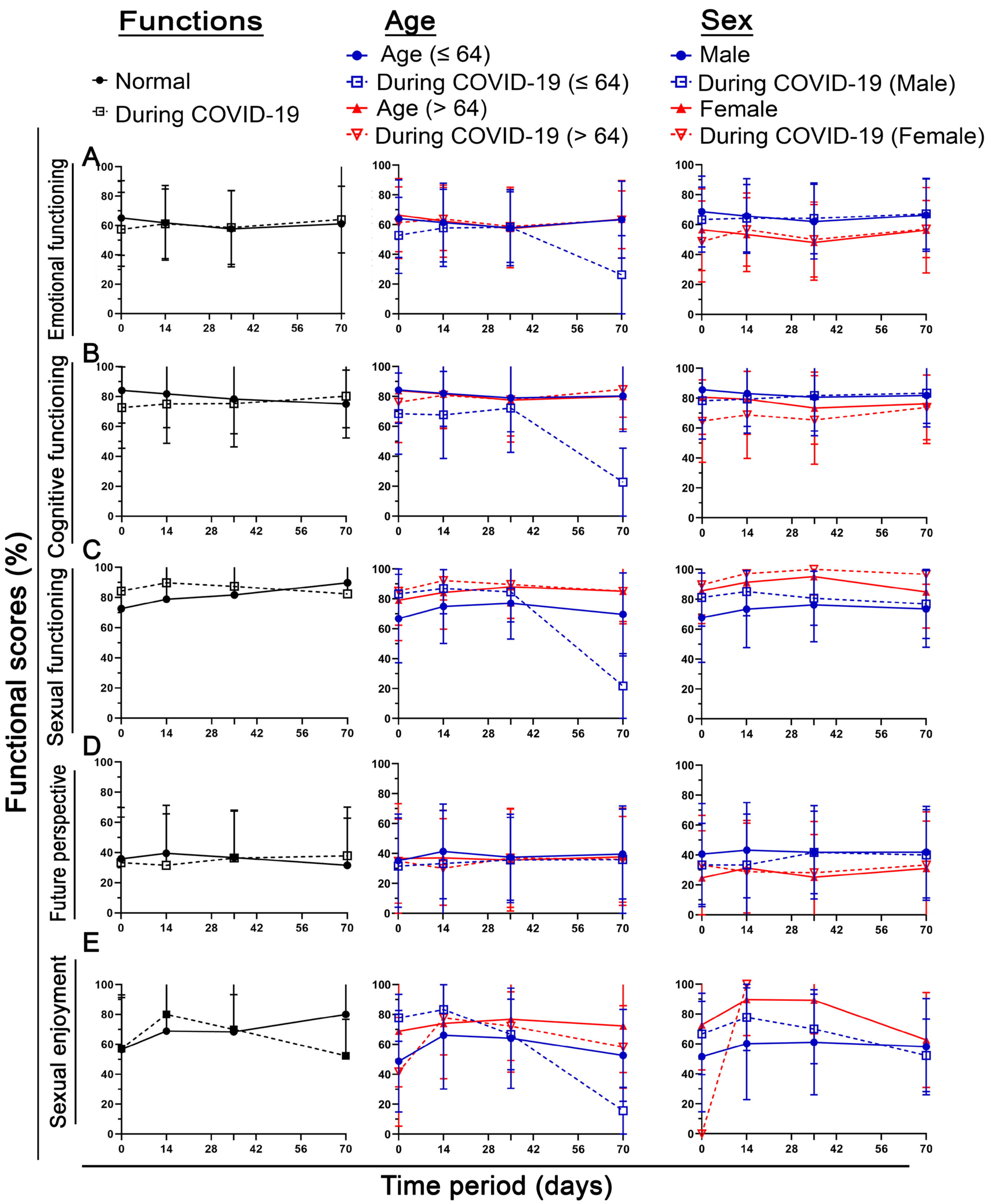

Figure S2. *Time course of different functional scores at day -1, day 14, day 35 and day 70. In the first column the entire cohort is separated into normal (before COVID-19) and during COVID-19. In the second column the cohort was divided in patients younger or 64 to older than 64. In the third column females and males of the cohort were compared for* ***(A)*** *emotional functioning,* ***(B)*** *cognitive functioning,* ***(C)*** *sexual functioning,* ***(D)*** *future perspective, and* ***(E)*** *sexual enjoyment.*

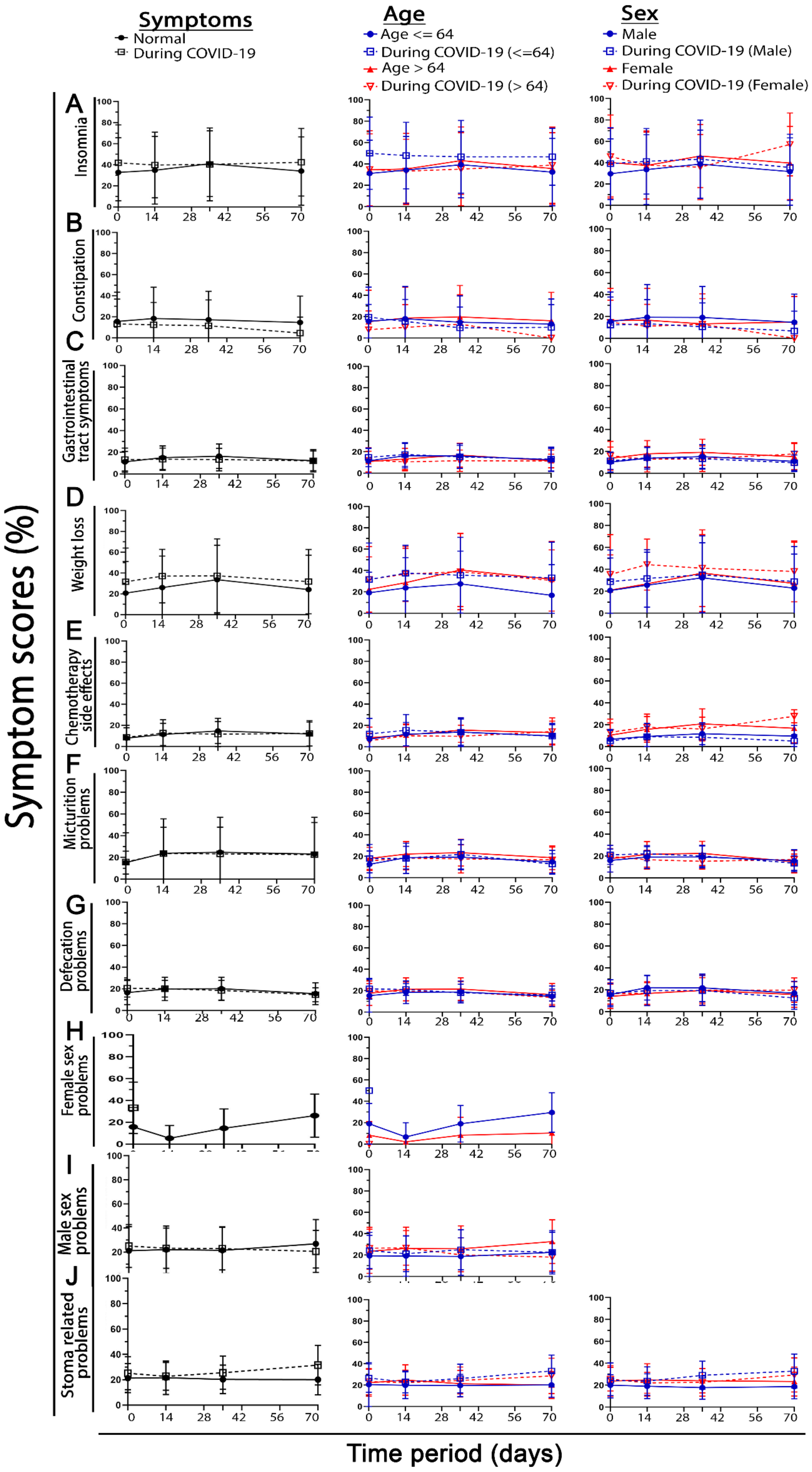
Symptoms
Normal
During COVID-19
Age
Age <= 64
During COVID-19 (<=64)
Age > 64
During COVID-19 (> 64)
Sex
Male
During COVID-19 (Male)
Female
During COVID-19 (Female)
A Insomnia
B Constipation
C Gastrointestinal tract symptoms
D Weight loss
E Chemotherapy side effects
F Micturition problems
G Defecation problems
H Female sex problems
I Male sex problems
J Stoma related problems
Symptom scores (%)
Time period (days)

Figure S3. *Time course of different symptom scores at day -1, day 14, day 35 and day 70. In the first column the entire cohort is separated into normal (before COVID-19) and during COVID-19. In the second column the cohort was divided in patients younger or 64 to older than 64. In the third column females and males of the cohort were compared for* ***(A)*** *insomnia,* ***(B)*** *constipation,* ***(C)*** *gastrointestinal tract symptoms,* ***(D)*** *weight loss,* ***(E)*** *chemotherapy side effects,* ***(F)*** *micturition,* ***(G)*** *defecation problems,* ***(H)*** *female sex problems,* ***(I)*** *male sex problems, and* ***(J)*** *stoma related problems at the different time periods at which the surveys took place, at day -1, day 14, day 35 and day 70.*
